# Monkey orange (*Strychnos spinosa* lam): a review of phytonutrients, bio-accessibility, value-added products, and potential as a functional food

**DOI:** 10.3389/fnut.2026.1875659

**Published:** 2026-07-28

**Authors:** Tyson Tebogo Mokgalabone, Semakaleng Mpai, Nontuthuko Rosemary Ntuli, Nhlanhla Mathaba, Ashwell Rungano Ndhlala

**Affiliations:** 1 Faculty of Science and Agriculture, University of Limpopo, Mankweng, South Africa; 2 Faculty of Science, Agriculture and Engineering, University of Zululand KwaDlangezwa, South Africa

**Keywords:** bio-accessibility, fermentation, phytonutrients, *Strychnos spinosa*, value-added products

## Abstract

**Introduction:**

*Strychnos spinosa* Lam. (monkey orange) is a drought-tolerant indigenous fruit tree distributed across sub-Saharan Africa, valued traditionally for nutrition and medicine but remaining underutilised in formal economies. This review synthesises literature on the fruit’s physicochemical properties, phytonutrient profile, value-added products, and bio-accessibility to evaluate its potential as a functional food.

**Methods:**

A systematic literature search was conducted following PRISMA guidelines across database using different keyword strings. Of 387 initial records identified, 75 studies met inclusion criteria and were included in the qualitative synthesis.

**Results:**

The pulp is characterised by high acidity (pH 2.5–4.0), soluble solids (12–22 °Brix), and pulp yield (30–60%). Nutritionally, it is rich in carbohydrates, dietary fibre, and minerals including potassium, magnesium, calcium, iron, and zinc. Phytochemical profiling revealed bioactive compounds such as chlorogenic acid, caffeic acid, rutin, and carotenoids, underpinning antioxidant, antimicrobial, and anti-inflammatory activities. Value-added products including juices, jams, fermented beverages, and dried rolls have demonstrated feasibility. However, a critical knowledge gap persists: while compositional analyses confirm high phytonutrient content, their bio-accessibility remains poorly characterised.

**Discussion:**

Factors including carotenoid lipophilicity, phenolic compound stability during digestion, and processing impacts on nutrient release have not been systematically quantified. Without integrating standardised bio-accessibility studies, the physiological relevance of *S. spinosa’s* phytonutrients cannot be validated, and commercialisation as a functional food remains speculative. Fermentation emerges as a promising traditional and scalable method to enhance nutrient bioavailability through phenolic modification, cell wall disruption, and reduction of anti-nutritional factors.

**Conclusion:**

This review recommends prioritising bio-accessibility investigations alongside fermentation optimisation to transform *S. spinosa* into a validated, climate-smart functional food for sub-Saharan Africa, supporting evidence-based product development, nutrition security, and rural economic opportunities.

## 1 Introduction

In developing countries, population growth, deforestation, and climate change are primary drivers of food and nutrition insecurity, largely due to inadequate access to sufficient, nutritious food (1). These challenges persist across Africa, where widespread poverty and malnutrition continue to affect a significant portion of the population (2). In this context, native edible fruits offer a promising solution: well-adapted to local environments, prodiving essential nutrients and contribute meaningfully to improving food security (3, 4). *Strychnos spinosa* Lam., commonly known as monkey orange fruit in English and Morapa in Sepedi, belongs to the Loganiaceae family (5). It is widely distributed across sub-Saharan Africa, including South Africa, Zimbabwe, and Botswana (5). The tree thrives in semi-arid climates and is commonly found in savannahs and woodlands. In South Africa, it predominantly grows in the Eastern Cape, Limpopo, KwaZulu-Natal, and Mpumalanga provinces (6). About 75 *Strychnos* species have been identified in Africa and around 20 species are edible (6–8). The most frequently consumed species in Southern Africa include *Strychnos innocua, Strychnos pungens*, and *Strychnos spinosa* (5, 7).

- The global functional food market has experienced significant growth, projected to reach USD 600 billion by 2030, driven by increasing consumer awareness of the link between diet and health (Grand View Research, 2024). Within this expanding market, underutilised indigenous fruits are gaining attention as sources of novel bioactive compounds and sustainable ingredients. *Strychnos spinosa* represents a promising candidate for inclusion in this market, with its drought tolerance, nutritional richness, and distinctive flavour profile offering competitive advantages for product development (9). Traditionally, the fruit has been consumed by rural communities as a source of nutrition and medicine. The tree has the capacity to yield a harvest even during drought, since it is well adapted to its local environment, while staple crops fail (10), which is an important characteristic for food and nutrition security, as this will enhance availability and productivity (11). Despite the notable yield of fruit biomass (12, 13), due to their limited availability, these native fruits remain underutilized and many communities continue to face challenges related to inadequate food access (14). Hence, fruits of this species remain confined to local consumption and has not ventured into commercial markets (8, 15).

Several rural communities have already embraced the creation of homemade fruit-based products using *S. spinosa* (5, 6). Indigenous uses of *S. spinosa* included direct fruit consumption; development of local and nutrient dense food products and drinks, such as fermented maize meal, fermented porridge, alcohol, juice, and jam; various medicines; as well as for homestead protection, livestock increase and firewood (6). *Strychnos spinosa* shows promising prospects for industrial utilization, particularly in the realm of producing fruit juice-based products (15). The fruits are rich in macronutrients, micronutrients, and dietary phytochemicals and have several health benefits (16). While the pulp demonstrates a rich phytochemical profile (17, 18) and the presence of these compounds alone does not confirm their physiological contribution without considering their bio-accessibility and bio-availability. And *S. spinosa* remains largely underexploited in formal agro-economies, classifying it as a neglected and underutilised species (NUS).

Previous reviews on *S. spinosa* have focused primarily on its ethnobotanical uses, phytochemical composition, and biological activities (19, 20). However, none have systematically addressed the critical gap between compositional richness and physiological relevance through bio-accessibility assessment. Furthermore, the potential of fermentation to enhance nutrient release and the practical pathways for commercialisation have received limited attention. This review distinguishes itself by: (i) integrating a critical focus on bio-accessibility as the missing link between composition and health benefits, (ii) synthesising value-added product development with specific emphasis on fermentation optimisation, (iii) providing a comprehensive comparative analysis of *S. spinosa* against other indigenous African fruits, and (iv) offering actionable recommendations for research and commercialisation. This will synthesise contemporary scientific literature on *S. spinosa* fruit, focusing on its physicochemical properties, phytonutrient profile, nutritional potential, and the bio-accessibility of its compounds, with the aim of providing a roadmap for its development as a validated, climate-smart functional food.

## 2 Literature search and data collection

**Figure 1:**
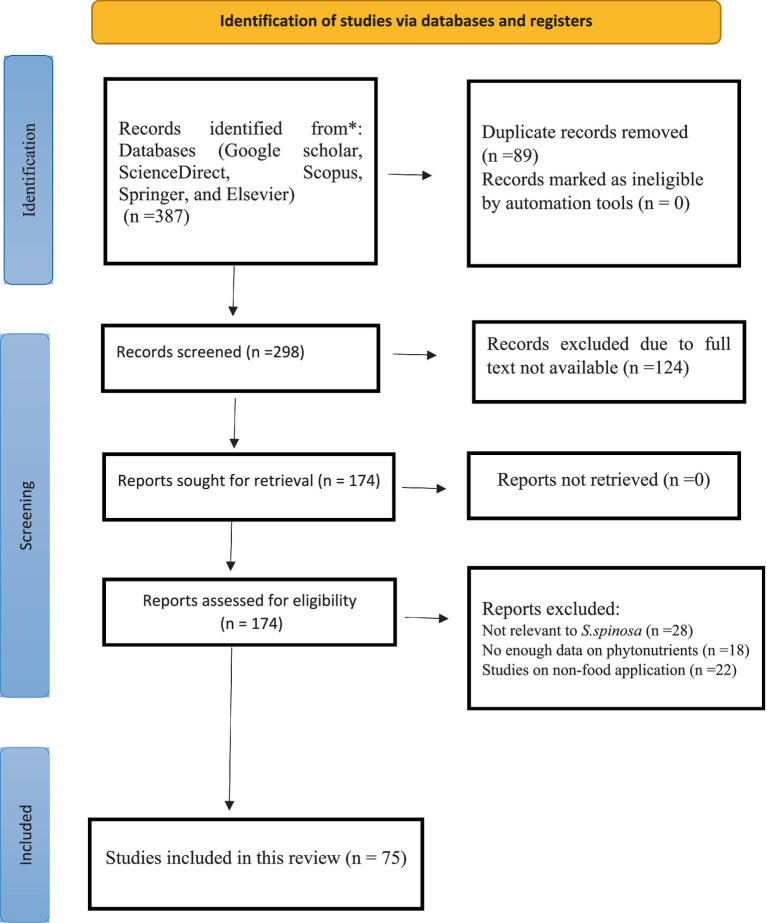
PRISMA Flowchart of literature identification.

To ensure methodological transparency, a systematic literature search was conducted following PRISMA (Preferred Reporting Items for Systematic Reviews and Meta-Analyses) guidelines (Figure 1). The literature search covered publications up to March 2026. Relevant research articles were gathered from online databases including Scopus, Web of Science, PubMed, Google Scholar, ScienceDirect, and SpringerLink, employing Boolean keyword strings combining “*Strychnos spinosa*” OR “monkey orange” with “phytonutrients” OR “phytochemicals” OR “antioxidants” OR “phenolics” OR “flavonoids” OR “carotenoids” and “bio-accessibility” OR “bioavailability” OR “digestion” and “processing” OR “fermentation” OR “value-added”.

The initial literature search yielded 387 records. Following removal of 89 duplicate records, 298 unique records were screened at the title level, resulting in 124 exclusions as not relevant to *S. spinosa* fruit, phytonutrients, or food processing. Abstract screening of the remaining 174 records resulted in 78 exclusions, including studies on other *Strychnos* species without clear relevance to *S. spinosa* (*n* = 28), studies on non-food applications (*n* = 22), studies with insufficient data on nutritional or phytochemical content (*n* = 18), and duplicate reporting of the same data (*n* = 10). Full-text review of the remaining 96 articles resulted in 21 exclusions due to non-English language (*n* = 8), insufficient quantitative data for synthesis (*n* = 7), retracted articles (*n* = 2), and inability to locate full text (*n* = 4). A total of 75 studies were included in the qualitative synthesis (Figure 1).

Inclusion criteria comprised: (i) peer-reviewed articles published in English, (ii) original research, reviews, and ethnobotanical studies on *S. spinosa* or closely related species, (iii) studies focusing on nutrition, phytochemistry, processing, value-addition, fermentation, and bio-accessibility, and (iv) articles with sufficient quantitative or qualitative data for synthesis. Exclusion criteria included: (i) non-English articles, (ii) studies not directly relevant to the fruit or its products, (iii) articles without sufficient data, and (iv) studies on other *Strychnos* species without clear relevance to *S. spinosa*. Preprints were not considered due to lack of peer review, and non-English studies were excluded due to resource constraints for translation and the predominance of English-language scientific literature on this topic. This language restriction is acknowledged as a limitation.

A meta-analysis was not conducted due to substantial heterogeneity among included studies, including differences in study design, geographic locations and ecological conditions, processing methods and analytical techniques, outcome measures, methodological quality, and completeness of reporting. Given this heterogeneity, a narrative synthesis was conducted, organizing findings by thematic categories.

## 3 Morphological features, geographical distribution and physicochemical attributes

**Figure 2:**
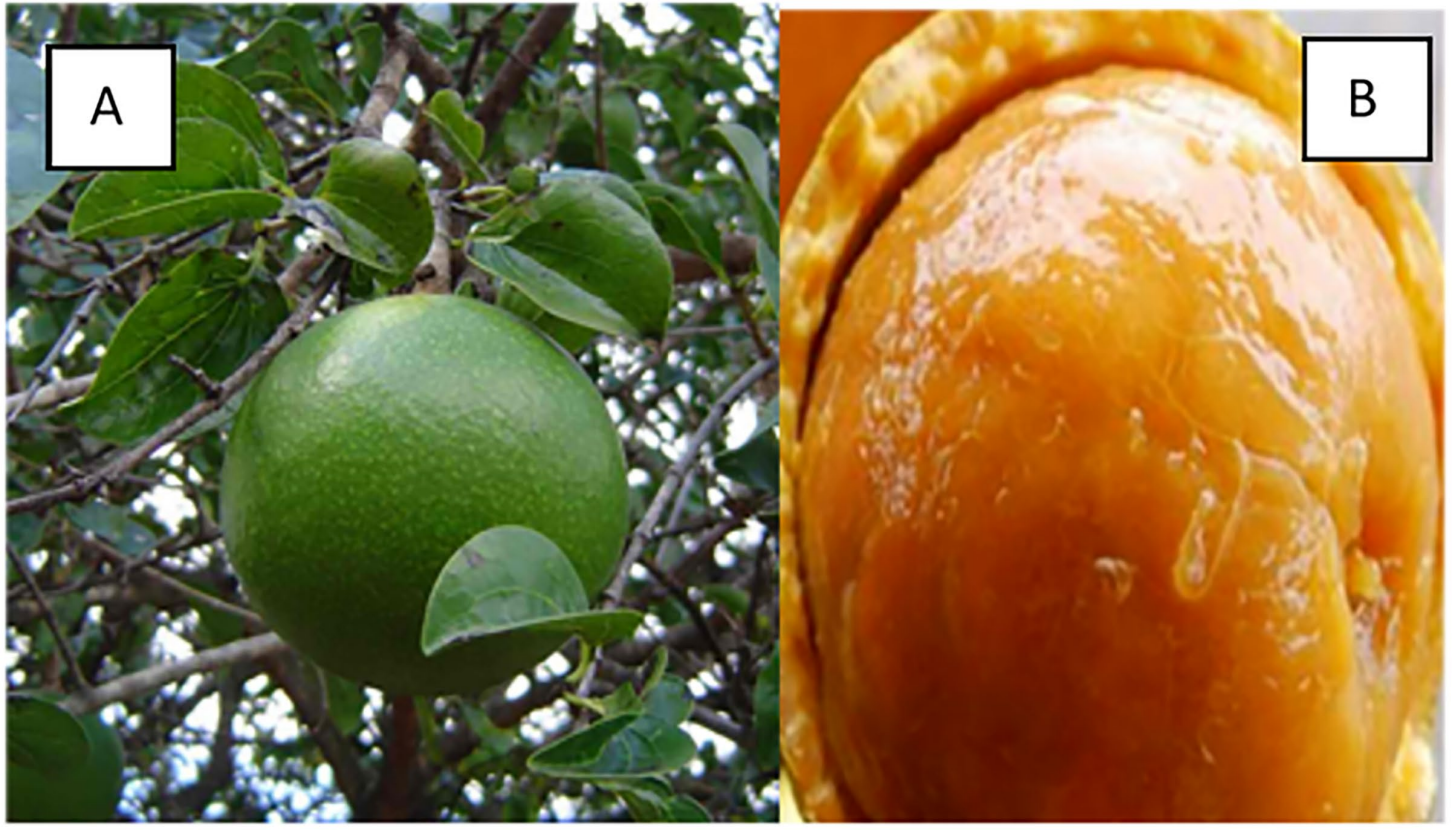
*Strychnos spinosa* showing **(A)** leaves and unripe fruit, and **(B)** ripe fruit with yellow pulp. Reproduced from Omotayo et al. (69). Licensed under CC BY 4.0.

Morphologically, *S.spinosa* is a shrubby deciduous plant that typically grows between 1 and 9 meters tall (21). It has a distinctive corky bark and characterized by the presence of straight and curved spines. As shown in Figure 2A the leaves are simple, oval-shaped, and glabrous (smooth), arranged in opposite pairs with axillary spines (22). The tree produces edible fruits with hard pericarp that are initially bright green in colour when unripe (Figure 2A), with a woody peel approximately 3–4 mm thick. As the fruit ripens, it turns yellow-brown (23). The shell’s thickness and hardness serve as a protective barrier against environmental stress and predators. The size of the fruit varies, ranging from 10 to 15 cm in diameter. The fruit contains flat seeds that are slightly similar in appearance to apricot seeds. The fruit has a yellow, edible, juicy, and sweet–sour pulp (Figure 2B) which may be due to the presence of high concentration of carotenoids (18, 21, 22).

**Figure 3:**
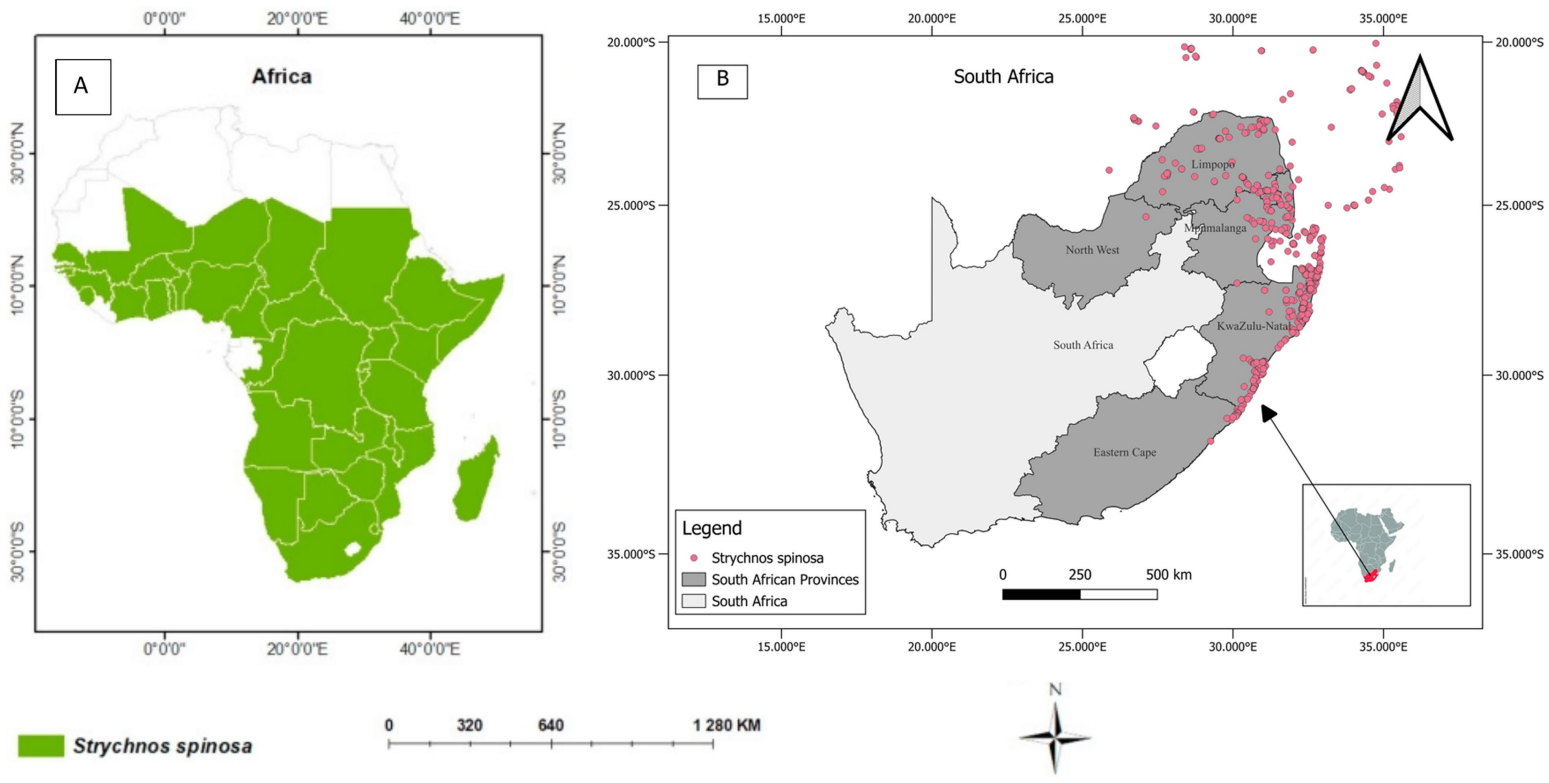
Geographic distribution of *Strychnos spinosa* Lam. in Africa. **Panel A:** Distribution across sub-Saharan Africa; **Panel B:** Distribution within South Africa, showing major provinces where the species occurs. Panel A is an adaptation from The International Plant Names Index and World Checklist of Vascular Plants 2026. Published on the Internet at http://www.ipni.org and https://powo.science.kew.org/. © Copyright 2023 World Checklist of Vascular Plants, licensed under CC BY 3.0. Panel B is reproduced from inaturalist.org, licensed under CC BY-NC 4.0.

The fruit tree is widely distributed across the continent, as mentioned above it grows well in various habitats including savannas, woodlands, and forests, and preferring well-drained soils (23–26). As indicated in Figure 3, the species is found in Southern Africa, including Angola, Botswana, Madagascar, Malawi, Mozambique, Namibia, South Africa, Swaziland, Zambia, and Zimbabwe, as well as in East Africa, including Ethiopia, Tanzania, and Uganda, and in West Africa, including Benin, Ghana, Guinea Bissau, and Nigeria (23, 27, 28). The species grows in various ecological zones, including savannah forests, open woodlands, and riverine fringes, and can thrive in both tropical and subtropical climates (21, 29, 30). Production data indicate that mature trees can yield up to 200–500 fruits per season, with fruit weights ranging from 50 to 300 g depending on genotype and growing conditions (24, 31). Estimated annual production in key regions suggests substantial biomass, although systematic production statistics remain limited due to the species’ underutilised status.

Research on physicochemical attributes of *S.spinosa* fruit provides essential data for post-harvest handling and processing. The pH of the pulp is generally acidic, ranging from 2.5 to 4.0 (Table 1), which contributes to its tart flavour and microbiological stability (32). Total soluble solids (TSS), primarily sugars, range from 12 to 22 °Brix, indicating a moderate to high sweetness potential (33). Titratable acidity (TA) is high, often dominated by organic acids like citric and malic acid, resulting in a low TSS/TA ratio that defines its characteristic sweet–sour taste profile (18). Pulp yield, a crucial parameter for processors, varies widely (30–60% of fresh fruit weight) (31). This variability is likely due to genetic differences and environmental factors affecting shell thickness. Studies have shown that environmental conditions, including rainfall patterns, soil nutrient status, and temperature, significantly influence fruit quality attributes (31). Genotype diversity within the species also contributes to variations in pulp yield, sugar content, and acidity. Future agronomic or breeding programs aimed at domestication must prioritise high-pulp-yielding genotypes to improve economic viability.

**Table 1:** Summary of physicochemical attributes of *S. spinosa* fruit pulp.

Attribute	Key compounds	References
Pulp Colour	Yellow to deep orange	Sivakumar et al. (18)
pH	2.5–4.0	Mokganya et al. (32)
Total Soluble Solids (°Brix)	12–22	Maseko et al. (33)
Titratable Acidity (%)	High (exact range varies)	Sivakumar et al. (18)
Pulp Yield (%)	30–60	Mamba et al. (31)

## 4 Chemical composition in *Strychnos spinosa* fruit

### 4.1 Nutritional composition and health-promoting potential of *Strychnos spinosa*

Nutritionally, *S. spinosa* fruits are rich in carbohydrates, crude protein, fats, fiber, and essential minerals, making them a valuable source of nutrients (9, 34, 35). The fruit of *S. spinosa* is a rich source of diverse nutrients at varying levels, which are comparable to concentrations in other indigenous and exotic fruits (9, 36, 37). The majority of nutrients existing in indigenous fruits are important for maintaining human health (9, 10, 36).

Proximate analysis (Table 2) revealed a composition high in moisture (70–80%) and carbohydrates (15–25%), with low fat (<1%) and protein (1–3%) content (9, 33). The low protein and fat content indicate that *S. spinosa* should be viewed as a complementary source of micronutrients, carbohydrates, and dietary fibre rather than a primary protein or lipid source. This positions the fruit well for dietary diversification strategies, where it can complement staple foods that are typically carbohydrate-rich but micronutrient-poor. It is a noteworthy source of dietary fibre, which aids digestive health and may contribute to glycaemic control and satiety.

**Table 2:** Nutritional composition of *Strychnos spinosa* fruit pulp.

Attribute	Key compounds	Key references
Moisture Content	70–80%	Maseko et al. (33)
Carbohydrates	15–25%	Maseko et al. (33)
Crude Protein	1–3%	Maseko et al. (33)
Crude Fat	< 1%	Maseko et al. (33)
Prominent Minerals	K, Mg, P, Ca, Zn, Fe	Mokganya et al. (32)
Dominant Sugars (HPLC)	Sucrose, Glucose. Fructose	Sivakumar et al. (18)

The mineral profile is a standout feature; the fruit is particularly rich in potassium, an essential electrolyte, and contains significant amounts of magnesium, phosphorus, calcium, and zinc (Table 2) comparable to the level found in commercial fruits such as apple (*Malus domestica*) and orange (*Citrus maxima*) (9, 32, 38, 39). Iron content is also appreciable, suggesting potential in addressing micronutrient deficiencies prevalent in its growing regions. The fruit’s high nutritional content underscores its potential to address malnutrition and improve dietary diversity in rural areas (27). The sugar composition, analysed via HPLC, typically showed a predominance of sucrose, followed by glucose and fructose (18). This profile contributes to its energy-giving properties. There are limited studies on amino acid profiles which indicates the presence of essential amino acids like leucine, valine, and phenylalanine, though not in complete protein-forming proportions, highlighting the fruit’s role as part of a balanced diet rather than a primary protein source (33). This positions the fruit not just as a food, but as a functional ingredient for dietary diversification and addressing hidden hunger.

### 4.2 Phytochemicals and biological activity associated with *Strychnos spinosa* fruit

Beyond the value of *S.spinosa* as a resilient fruit crop, several studies have revealed that different plant parts particularly the leaves, bark, seeds and fruit contain diverse phytochemicals with relevant biological properties. Phytochemicals are non-nutritive, secondary metabolites, for example phenolic acids, flavonoids and carotenoids (40, 41). These compounds are associated with various health benefits, which includes reducing the risk of chronic diseases like cardiovascular conditions, neurodegenerative diseases, and others (42). Over the past two decades, interest in *S.spinosa* has expanded due to evidence of antioxidant, antimicrobial, anti-inflammatory, antidiabetic and antiparasitic activities (19, 39). The biological properties of *S. spinosa* extracts are strongly influenced by their phytochemical richness.

Analysis of the fruit extracts has revealed the presence of diverse phytochemicals (Table 3). *Strychnos spinosa* exhibits total phenolic content (40 mg GAE/100 g) comparable to marula (*Sclerocarya birrea*) (35–55 mg GAE/100 g) and wild medlar (*Vangueria infausta*) (45–60 mg GAE/100 g), but substantially lower than baobab (*Adansonia digitata*) (120–150 mg GAE/100 g) and ber (*Ziziphus mauritiana*) (80–110 mg GAE/100 g). This intermediate position suggests that while *S. spinosa* is not the most phenolic-rich indigenous fruit, its phenolic content is still nutritionally relevant. Notably, *S. spinosa* demonstrates higher flavonoid content (55 mg CE/100 g) compared with baobab (25–40 mg CE/100 g) and marula (30–45 mg CE/100 g), indicating that flavonoids are a particularly significant phytochemical class in this fruit. This flavonoid richness is important because flavonoids exhibit potent antioxidant, anti-inflammatory, and cardioprotective properties (43). The proanthocyanidin content of *S. spinosa* (0.47% leucocyanidin equivalents) is comparable to baobab (0.30–0.45%) and wild medlar (0.25–0.40%). Proanthocyanidins are polymeric flavonoids with strong protein-binding properties that contribute to astringency and may offer health benefits including antimicrobial activity and blood sugar regulation (68). However, the levels observed are unlikely to pose anti-nutritional concerns, distinguishing *S. spinosa* from other *Strychnos* species that may contain higher alkaloid concentrations.

**Table 3:** Phytochemical composition of *Strychnos spinosa* fruit pulp compared with selected indigenous African fruits.

Phytochemical category	*Strychnos spinosa*	*Adansonia digitata*	*Sclerocarya birrea*	*Ziziphus mauritiana*	*Vangueria infausta*	References
Total Phenolics (mg GAE/100 g)	40	120–150	35–55	80–110	45–60	Nhukarume et al. (17) and Moyo et al. (57)
Total Flavonoids (mg CE/100 g)	55	25–40	30–45	50–70	35–50	Nhukarume et al. (17), Moyo et al. (57), Tahir et al., (66), and Mwaura et al. (58)
Proanthocyanidins (% leucocyanidin eq.)	0.47	0.30–0.45	0.20–0.35	0.55–0.80	0.25–0.40	Isa et al. (59), Moyo et al. (57), Tahir et al. (66), Dragone et al., (64) and Mwaura et al. (58)
Carotenoids (μg/g)	Lutein + β-carotene (not quantified)	Not detected	2–5	3–8	1–3	Isa et al. (59), Moyo et al. (57), and Tahir et al. (66)
Major Phenolic Compounds Identified	Chlorogenic acid, caffeic acid, rutin, secoxyloganin	Procyanidins, epicatechin, gallic acid	Gallic acid, ellagic acid, quercetin, myricetin	Catechin, epicatechin, rutin, quercetin	Chlorogenic acid, caffeic acid, rutin	Sitrit et al. (27), Moyo et al. (57), and Ngadze et al. (5)
Major Volatiles	trans-isoeugenol, eugenol, chavicol, vanillin	No significant volatiles	Terpenes, esters, alcohols	Aldehydes, alcohols	Esters, terpenoids	Isa et al. (59), Moyo et al. (57), Tahir et al. (66), and Ngadze et al. (5)
Antioxidant Activity (DPPH IC₅₀, μg/mL)	33 (fruit extract)	25–40	45–60	30–50	50–70	Isa et al. (59) and El Bilali et al. (60)

A key distinguishing feature of *S. spinosa* is the presence of secoxyloganin, an iridoid glycoside not reported in the other indigenous fruits surveyed. This compound, identified by Nhukarume *et al*. (17), has been associated with anti-inflammatory and neuroprotective activities in other plant species (44). The presence of chlorogenic and caffeic acid places *S. spinosa* in a similar phenolic profile group as wild medlar, while differing from marula (dominated by gallic and ellagic acids) and baobab (dominated by procyanidins). These qualitative differences are important because different phenolic compounds exhibit distinct bioavailability, metabolic pathways, and biological activities. For example, chlorogenic acid is known for its hypoglycaemic effects, while caffeic acid exhibits potent anti-inflammatory activity (67). Among the five fruits compared, *S. spinosa* is notable for containing significant levels of carotenoids (lutein and *β*-carotene), whereas baobab pulp lacks detectable carotenoids, and marula and *Ziziphus mauritiana* contain only low levels. The yellow to deep orange colour of ripe *S. spinosa* pulp is a direct visual indicator of carotenoid accumulation. This positions *S. spinosa* as a potentially valuable source of provitamin A carotenoids in regions where vitamin A deficiency is prevalent (18). However, the bioaccessibility of these carotenoids remains a critical consideration, with lipid co-digestion being essential for effective absorption.

The volatile profile of *S. spinosa* is exceptionally rich and distinctive, dominated by phenylpropanoids (eugenol, trans-isoeugenol, chavicol) and vanillin, which together produce a spicy, sweet, and aromatic character (27). This volatile profile is unique among the compared indigenous fruits; marula produces primarily terpenes and esters, while *Ziziphus mauritiana* produces aldehydes and alcohols. The presence of vanillin, a high-value flavour compound, is particularly noteworthy and suggests commercial potential for natural flavour extraction. The antioxidant activity of *S. spinosa* fruit extracts (DPPH IC₅₀ of 33 μg/mL) are comparable to baobab (25–40 μg/mL) and *Ziziphus mauritiana* (30–50 μg/mL), and superior to marula (45–60 μg/mL) and wild medlar (50–70 μg/mL). This relatively high antioxidant capacity, despite moderate total phenolic content, suggests that *S. spinosa* contains particularly potent radical-scavenging compounds, possibly due to the specific arrangement of hydroxyl groups on its flavonoid and phenolic acid constituents.

The most promising phytochemicals in *S. spinosa* for functional food applications are chlorogenic acid (hypoglycaemic potential), rutin (anti-inflammatory and cardioprotective), carotenoids (provitamin A activity), and the distinctive iridoid secoxyolganin (neuroprotective potential). These compounds, combined with the fruit’s high flavonoid content and favourable antioxidant capacity, support the development of *S. spinosa* as a functional food ingredient targeting metabolic health, inflammation, and oxidative stress-related conditions. However, caution is warranted in extrapolating *in vitro* results to *in vivo* health benefits; future research must validate these activities through well-designed clinical studies.

### 4.3 History of human consumption and toxicological considerations

*Strychnos spinosa* has a long history of human consumption across sub-Saharan Africa, with archaeological and ethnographic evidence indicating its use for centuries by indigenous communities (6, 23). The fruit has been traditionally consumed fresh, dried, or processed into various products, forming part of the seasonal diet and serving as a food reserve during periods of scarcity. Traditional knowledge has guided the safe use of the fruit, including the practice of consuming only the ripe pulp and avoiding the seeds and unripe fruit. From a toxicological perspective, the fruit pulp of *S. spinosa* is generally regarded as safe based on centuries of traditional consumption without reported acute toxicity (39). However, it is important to note that the seeds and other plant parts contain *strychnine* and related indole alkaloids, which are neurotoxic compounds (19). These alkaloids are concentrated in the seeds, bark, and roots, with minimal transfer to the pulp (45). Traditional practices of removing seeds during processing provide an important safety measure. Systematic toxicological studies specifically on the fruit pulp remain limited. No acute or sub-chronic toxicity studies have been published, and allergenicity assessments have not been conducted. Furthermore, potential interactions with medications have not been investigated. As commercial development of *S. spinosa* products progresses, systematic toxicological evaluations will be necessary to support regulatory approval and consumer safety. These should include acute and sub-chronic toxicity studies, genotoxicity assessments, allergenicity testing, and evaluation of potential drug interactions.

## 5 Value-added products from *Strychnos spinosa* and their phyto-nutritional benefits

### 5.1 Traditional processing and household uses of *Strychnos spinosa* fruit

*Strychnos spinosa* is a neglected but nutritionally promising indigenous fruit of southern and eastern Africa. Although widely gathered and consumed locally, formal development of value-added food products has been limited. Literature describing traditional uses and pilot processing trials indicates that the fruit pulp can be converted into a variety of products including non-alcoholic juices, jams, syrups, fruit rolls and fermented beverages (local wines and mahewu-style products) and that these products may retain appreciable amounts of vitamins, minerals and phenolic antioxidants that underpin the fruit’s phyto-nutritional value (20, 24, 27) (Table 4). Across rural communities the fruit is eaten fresh when ripe, sun-dried for later use, or processed into simple household products. Traditional small-scale processing commonly yields reconstituted pulp drinks, porridge enrichments, fruit rolls and preserves (5). Ethnographic and survey studies report that *Strychnos* pulp is used as an ingredient in porridge and the fermented cereal beverage mahewu, and that community-level fermentation to make local wines is practiced in some areas (24, 46). More formal product development work has demonstrated feasibility for juice, jam, jelly and muffins made from monkey orange, with sensory trials indicating consumer acceptance comparable with other indigenous fruit products when suitable processing parameters are used (47).

Jam and jelly production converts the pulp into a shelf-stable product that concentrates sugars and soluble solids while conserving some micronutrients and phytochemicals. Pilot studies and value-addition experiments for *S. spinosa* have followed conventional pectin/sugar/gelling protocols and reported acceptable sensory attributes for jams and jellies made from mixed indigenous fruits (47). From a nutritional perspective, thermal processing used in jam manufacture reduces vitamin C but can concentrate minerals, fibre-associated components and phenolic compounds bound to cell walls; consequently, jams may provide an accessible source of iron, calcium, and antioxidant polyphenols, albeit with reduced vitamin C compared with fresh pulp (13, 27). Product optimization such as fermentation could help preserve labile phytonutrients while delivering acceptable shelf-life.

Juices and reconstituted drinks are commonly produced at household and small commercial scale. The pulp yields a tangy, vitamin-C rich beverage (20, 27). Laboratory analyses consistently report high total soluble solids and appreciable vitamin C contents in ripe fruit, together with significant levels of phenolic antioxidants (13, 20). Processing challenges include particulate removal (seeds are numerous and hard), astringency from seed- or peel-associated compounds, and microbial stability. Simple improvements such as enzymatic clarification, pasteurisation, and hygiene controls have been shown to produce shelf-stable juices that retain a meaningful portion of the fruit’s antioxidant capacity (24). Reconstituted and blended juices (mixed with more neutral fruit juices) improve palatability and may increase consumer acceptance while providing a vehicle for micronutrient delivery.

Fermented beverages represent an important avenue for value addition because fermentation can transform flavour, increase caloric yield, and in some cases enhance bioavailability of micronutrients. Ethnobotanical records and processing studies document small-scale fermentation of *Strychnos* pulp to produce wines and fermented cereal drinks spiked with *S.spinosa* (mahewu-type), and government research in parts of Malawi and Zambia has previously trialled *Strychnos* species for wine production (24, 46). The fermentation kinetics, sugar profile, and nutrient release during *Strychnos* fermentations are not extensively documented in peer-reviewed literature, but available reports indicate that adequate sugar content and fermentable substrates are present in ripe pulp to produce traditional wines and beers (24, 27). Fermentation may modify phyto-nutrition in several ways. Microbial metabolism can reduce anti-nutritional factors and astringency, liberate bound phenolics, and increase folate and B-vitamin levels depending on the microbial consortia used (general fermentation literature). However, uncontrolled fermentations risk production of off-flavours, inconsistent alcohol yields and contamination; thus, standardised starter cultures and process control are recommended for commercialisation (24).

**Table 4:** Value-added products from *Strychnos spinosa* and their phyto-nutritional potential.

Products category	Specific examples	Phyto-nutrient insights	References
Jam and jelly	Jam, jellies	Concentrates sugars and soluble solids, provides an accessible source of antioxidant polyphenol	Cheikhyoussef et al. (47), Sitrit et al. (27), Ngemakwe (13), and Simbine-Ribisse et al. (61)
Juices and Beverages	Non-alcoholic juices, Blended beverages	Vitamin C-rich beverage, Contains high total soluble solids and phenolic antioxidants.	Sitrit et al. (27), Ngadze et al. (5), Omotayo and Aremu (20), and Ngemakwe (13)
Fermented Beverages	Traditional wines, mageu	Flavour enhancement, increase caloric yield, and enhance micronutrient bioavailability.	Mwamba (46), Ngadze et al. (5), and Sitrit et al. (27)
Dried and Other Products	Fruit rolls, Sun-dried pulp, Porridge enrichments	Increased energy density and mineral concentration.	Ngadze et al. (5), Cheikhyoussef et al. (47), and Simbine-Ribisse et al. (61)
General Nutritional Profile	Fresh Pulp	Rich in carbohydrates, dietary fibre, appreciable protein, Vitamin C, minerals (calcium, iron, potassium), and phenolic antioxidants.	Sitrit et al. (27), Ngemakwe (13), and Omotayo and Aremu (20)

### 5.2 Phyto-nutritional profiling of *Strychnos spinosa* products

Overall nutritional profiling studies place *S. spinosa* among nutrient-dense indigenous fruits: the pulp contains carbohydrates, dietary fibre, appreciable protein when compared with many other wild fruits, vitamin C, and minerals such as calcium, iron and potassium, together with a range of phenolic antioxidants (13, 20, 27). Processing alters this profile: jams and dried products have higher energy density and mineral concentration but lower vitamin C; juices retain vitamin C when minimally processed; fermentation can enhance some B-vitamins and reduce undesirable constituents, while distillation removes most non-volatile nutrients. Thus, product selection should align with nutritional goals: juices for vitamin C delivery; jams and rolls for energy and mineral provisioning; fermented products for caloric and potential probiotic benefits where safe starters are used. Value-added products from *S. spinosa* which include jams, juices, fermented beverages and dried rolls are feasible and can deliver significant phyto-nutritional benefits, particularly in vitamin C, minerals and phenolic antioxidants. Processing choices determine which nutrients are conserved or concentrated. *S. spinosa* is a promising candidate for nutritional diversification and rural value-addition strategies in sub-Saharan Africa.

## 6 Fermentation of *Strychnos spinosa*

Fermented products from *S. spinosa* are traditionally produced through spontaneous fermentation by wild yeasts and lactic acid bacteria naturally present on fruit surfaces (48), with *Saccharomyces cerevisiae* identified as the ideal fermentative yeast for alcoholic beverage production due to its ethanol tolerance and efficient sugar conversion (49). Survey data from smallholder farmers in Zimbabwe revealed that *S. spinosa* pulp is used as a functional ingredient in fermented porridge and the fermented cereal beverage by 25% of respondents, while community-level fermentation for traditional wine production is practised in some areas (24). In KwaZulu-Natal, South Africa, 97% of respondents reported using *S. spinosa* for fermented maize meal (umBhantshi), with additional uses including amaHewu (fermented porridge) and alcoholic beverages (6, 63). Traditional processing constraints include seed-flesh separation, long processing times, pulp viscosity, and phase separation during storage, which impact product consistency (24)0.6.1 Mechanisms of Phytonutrient Modification During Fermentation.

The fermentation of indigenous fruits enhances phyto-nutritional profile through several interconnected biochemical mechanisms. Microbial enzymes such as *β*-glucosidases, esterases, and decarboxylases actively modify phenolic compounds by hydrolyzing glycosylated forms like the flavonoid rutin into more bioaccessible aglycones, while also converting chlorogenic acid into caffeic acid derivatives with potentially greater anti-inflammatory activity (37). Concurrently, the microbial activity and organic acids produced during fermentation degrade cell wall polysaccharides particularly pectin, cellulose, and hemicellulose thereby disrupting the fruit matrix and facilitating the release of bound phytochemicals, including phenolic compounds and carotenoids, for improved digestive extraction (50). This process also reduces anti-nutritional factors such as phytates and tannins, which otherwise chelate minerals, thereby enhancing the bioaccessibility of essential minerals like iron, calcium, and zinc. Furthermore, fermentation generates new bioactive metabolites, including B-vitamins, organic acids, and exopolysaccharides, with related studies on lactic acid fermentation of similar fruits showing increased folate and B-vitamin levels depending on the microbial consortia employed, thus contributing synergistic antioxidant effects to the final product.

The fruit’s naturally high soluble solids (12–22 °Brix) provide adequate fermentable substrates (33). However, uncontrolled spontaneous fermentation results in inconsistent product quality between batches, necessitating the development of standardised starter cultures and optimised process controls for commercialisation (51). Recent studies on controlled fermentation of indigenous fruits have demonstrated the potential of selected starter cultures, including specific Lactobacillus strains for lactic acid fermentation and Saccharomyces strains for alcoholic fermentation (52, 53, 65). For *S. spinosa*, the development of regionally adapted starter cultures could improve product consistency, reduce fermentation time, and enhance the bioavailability of nutrients and bioactive compounds. Furthermore, the probiotic potential of fermented *S. spinosa* products warrants investigation, as lactic acid bacteria associated with fruit fermentations may offer health benefits beyond the nutritional content of the fruit itself. The positive consumer perception of fermented *Strychnos* products and reported health benefits, including improved weight gain and disease resistance in children and immune-compromised individuals (24), present opportunities for value addition and nutrition security enhancement, provided technological solutions for sensory quality and shelf-life stability are developed (6).

## 7 The imperative for bio-accessibility studies of *Strychnos spinosa* phytonutrients

Bio-accessibility refers to the fraction of a compound that is released from the food matrix in the gastrointestinal tract and becomes available for intestinal absorption (54). It is important to distinguish bio-accessibility from bioavailability and health benefits. The fraction of a compound released from the food matrix during digestion and available for intestinal absorption. This is a prerequisite for, but not equivalent to, bioavailability. Understanding the bio-accessibility of phytonutrients in *S. spinosa* is critical for determining its nutritional value and potential health benefits, especially given the rising interest in indigenous African fruits as sources of natural antioxidants for nutritional and functional food applications (20, 55).

### 7.1 Knowledge gap and research imperative

While compositional analyses confirm the pulp is rich in carotenoids, phenolic acids, and flavonoids (17, 18), the mere presence of these compounds does not guarantee their physiological benefit. Its high phenolic and carotenoid content (27, 55, 62) suggests that the fruit may provide substantial dietary antioxidants if these compounds survive digestion and are effectively solubilised for uptake. For instance, the lipophilic carotenoids essential for vitamin A activity require dietary lipids for emulsification and incorporation into mixed micelles to become bio-accessible, a process well-documented in other fruits but not yet quantified for *S. spinosa* (18). Similarly, the complex phenolic compounds may undergo significant chemical transformation or degradation in the gastrointestinal tract, altering their antioxidant potential (56).

Therefore, conducting standardized *in vitro* bio-accessibility studies on both fresh pulp and processed products (juices, jams, fermented beverages) is not merely an academic exercise but a fundamental necessity. These studies are crucial for two primary reasons: first, to scientifically validate traditional and contemporary claims regarding the fruit’s health benefits by bridging the gap between composition and physiological efficacy; and second, to inform intelligent value-addition strategies. By identifying how processing techniques such as fermentation, thermal treatment, or blending with lipid sources impact nutrient release and stability, researchers and food technologists can optimise product formulations to maximise the delivery of bio-accessible nutrients (33). Ultimately, without this crucial bio-accessibility data, efforts to commercialise *S. spinosa* as a functional food or nutraceutical ingredient remain speculative, potentially undermining its economic potential and its contribution to combating malnutrition and oxidative stress-related diseases in the regions where it grows. This underscores the need for *in vitro* bio-accessibility studies to become a standard part of the evaluation of *S. spinosa* product formulations, moving beyond simple compositional analysis.

## 8 The importance of this study

This comprehensive synthesis is critically important for three primary reasons. First, it addresses a significant knowledge gap by systematically collating fragmented research on *S. spinosa*, transforming isolated findings into a coherent evidence base that underscores its nutritional and economic value. Second, in the context of climate change and persistent food insecurity, this review highlights *S. spinosa* as a climate-smart, neglected crop that can diversify diets and farming systems. Third, and most crucially, it identifies the disconnect between nutrient composition and bio-accessibility as the fundamental barrier to realizing its health benefits. While numerous studies report on what the fruit *contains*, there is a stark lack of research on what the body can actually absorb and use. This review makes the explicit case that without investigating the digestive fate of its phytonutrients particularly through processing methods like fermentation, the true functional potential of *S. spinosa* remains unknown and its promotion as a superfood is premature. The roadmap provided for future research addresses this gap and guides the development of evidence-based functional food products.

## 9 Conclusion and recommendations

### 9.1 Conclusion

*Strychnos spinosa* is a resilient, drought-tolerant indigenous fruit tree with substantial potential to enhance food security, nutrition, and rural economies in sub-Saharan Africa. This review consolidates evidence that the fruit’s pulp is a rich source of essential macro- and micronutrients notably carbohydrates, dietary fibre, potassium, magnesium, and iron and a diverse array of bioactive phytochemicals, including phenolic acids such as chlorogenic and caffeic acid, flavonoids, and carotenoids. These compounds underpin its demonstrated *in vitro* antioxidant, antimicrobial, and anti-inflammatory activities, aligning with its traditional uses. The fruit’s physicochemical properties, including high acidity (pH 2.5–4.0), significant soluble solids (12–22 °Brix), and a robust hard shell, present both opportunities and challenges for post-harvest handling and processing. Successful value-addition pathways, including juices, jams, fermented beverages, and dried products, have been piloted, demonstrating feasibility and consumer acceptability. Fermentation, in particular, emerges as a promising traditional and scalable method to enhance flavour, preserve the fruit, and potentially improve the bioavailability of certain nutrients through specific mechanisms including phenolic modification, cell wall disruption, and reduction of anti-nutritional factors.

However, a critical gap exists between the documented high content of phytonutrients and the understood bio-accessibility. While the fruit is rich in antioxidants, their stability through gastrointestinal digestion, the essential role of dietary lipids for carotenoid micellisation, and the impact of processing on nutrient release remain poorly quantified. Furthermore, the spontaneous microbial ecology of traditional fermentations requires characterisation and optimisation to ensure product safety, consistency, and enhanced nutritional quality. Bridging these knowledge gaps is essential to transform *S. spinosa* from a locally appreciated, underutilised species into a validated, commercially viable, and health-promoting functional food.

### 9.2 Recommendations

Based on the comprehensive synthesis provided in this review, the following recommendations are proposed to advance research, development, and commercialisation of *S. spinosa*:

Research priorities:

- Standardised bio-accessibility studies: Conduct systematic *in vitro* digestion studies to quantify the bio-accessibility of key phytochemicals from fresh pulp and processed products.
- Processing optimisation: Evaluate the impact of different processing techniques on nutrient retention and bio-accessibility, to identify optimal processing parameters for specific nutritional targets.
- Fermentation characterisation: Systematically characterise the microbial ecology of traditional fermentations and develop regionally adapted starter cultures to ensure product consistency and safety.
- Pharmacokinetic studies: Conduct human intervention studies to assess the bioavailability and health effects of *S. spinosa* products following validation of bio-accessibility.
- Genotype selection: Identify high-performing genotypes with superior nutritional profiles, pulp yield, and processing characteristics for domestication and breeding programmes.

Product development and commercialisation:

- Consumer preference studies: Conduct market research to identify consumer preferences and willingness to pay for *S. spinosa* products in different regions.
- Value chain development: Develop sustainable value chains connecting smallholder farmers to processors and markets, with appropriate quality standards and certification schemes.
- Policy support: Engage with policymakers to recognise *S. spinosa* in national food security and agricultural diversification strategies, and to provide support for small-scale processing enterprises.
